# Synchronizing with the rhythm: Infant neural entrainment to complex musical and speech stimuli

**DOI:** 10.3389/fpsyg.2022.944670

**Published:** 2022-10-21

**Authors:** Chiara Cantiani, Chiara Dondena, Massimo Molteni, Valentina Riva, Caterina Piazza

**Affiliations:** ^1^Child Psychopathology Unit, Scientific Institute, IRCCS Eugenio Medea, Lecco, Italy; ^2^Bioengineering Lab, Scientific Institute, IRCCS Eugenio Medea, Lecco, Italy

**Keywords:** neural entrainment, infants, music, speech, EEG, steady-state evoked potentials (SS-EP)

## Abstract

Neural entrainment is defined as the process whereby brain activity, and more specifically neuronal oscillations measured by EEG, synchronize with exogenous stimulus rhythms. Despite the importance that neural oscillations have assumed in recent years in the field of auditory neuroscience and speech perception, in human infants the oscillatory brain rhythms and their synchronization with complex auditory exogenous rhythms are still relatively unexplored. In the present study, we investigate infant neural entrainment to complex non-speech (musical) and speech rhythmic stimuli; we provide a developmental analysis to explore potential similarities and differences between infants’ and adults’ ability to entrain to the stimuli; and we analyze the associations between infants’ neural entrainment measures and the concurrent level of development. 25 8-month-old infants were included in the study. Their EEG signals were recorded while they passively listened to non-speech and speech rhythmic stimuli modulated at different rates. In addition, Bayley Scales were administered to all infants to assess their cognitive, language, and social-emotional development. Neural entrainment to the incoming rhythms was measured in the form of peaks emerging from the EEG spectrum at frequencies corresponding to the rhythm envelope. Analyses of the EEG spectrum revealed clear responses above the noise floor at frequencies corresponding to the rhythm envelope, suggesting that – similarly to adults – infants at 8 months of age were capable of entraining to the incoming complex auditory rhythms. Infants’ measures of neural entrainment were associated with concurrent measures of cognitive and social-emotional development.

## Introduction

When listening to music, most people spontaneously move or clap following the rhythm. This happens because we are sensitive to periodic metric pulses, referred to as beats. Interestingly, even young children and preverbal infants are sensitive to rhythm and are able to detect the beat in music (Phillips-Silver and Trainor, 2005; Winkler et al., 2009). In the first year of life, infants already display spontaneous engagement and increased rhythmic movements and vocalizations in response to music and rhythmic patterns, although these movements are still not yet temporally modulated by the beat (Zentner and Eerola, 2010; Fujii et al., 2014; Rocha and Mareschal, 2017). These skills typically develop during the second year of life, with children starting to exhibit some tempo flexibility and synchronization (Zentner and Eerola, 2010; Fujii et al., 2014; Yu and Myowa, 2021). Further improvements are seen at older ages (Kirschner and Tomasello, 2009; Yu and Myowa, 2021). In the present study, we investigate neural entrainment to complex musical and speech stimuli in a sample of 8-month-old infants, in order to explore whether infants at this young age are able to synchronize at the neural level with the beat/meter of the stimuli or at least with the perceptual elements detectable in the sound.

Increased evidence suggests that the capacity for behavioral rhythmic sensorimotor synchronization is supported by neural entrainment mechanisms, such as cortical oscillations at specific frequencies. Neural entrainment is defined as the process whereby brain activity, and more specifically neuronal oscillations measured by electroencephalography (EEG), synchronizes with external (exogenous) stimulus rhythms. In the auditory modality, neural entrainment has been mainly investigated in response to two types of dynamic and rhythmic stimuli: speech and music (e.g., Nozaradan et al., 2011, 2012, 2018; Ding and Simon, 2012; Nozaradan, 2014; Tierney and Kraus, 2015; Ding et al., 2016; Zhou et al., 2016; Stupacher et al., 2017; Tal et al., 2017; Lenc et al., 2018; Jin et al., 2020). In these studies, low-frequency (< 6 Hz) neural entrainment has been reliably observed for both physical and abstract properties of the stimuli, such as the rhythms of musical beats and some linguistic constituents. For example, in response to simple musical rhythms Nozaradan et al. (2011) recorded entrained responses both to the existing beat frequency and to an imaginary meter frequency. Similarly, in response to speech stimuli, neural entrainment is typically recorded not only to low-level linguistic units but also to phrasal and sentential units (Ding et al., 2016, 2017). These pieces of evidence suggest that low-frequency neural entrainment may play a role not only in tracking speech and rhythmic sounds but also in parsing their temporal structures and extracting high-level chunks (Jin et al., 2020). They also suggest that neural entrainment might not be exclusively stimulus-driven, but additionally includes the contribution of an internally generated (endogenous) oscillator synchronized to the exogenous stimulation (Tal et al., 2017) and with a functional role in its processing (Obleser and Kayser, 2019).

The use of periodic sequences of stimulation and the recording of Steady-State Evoked Potentials (SS-EPs) has several advantages for the study of early neurocognitive development in infancy, as recently argued by Kabdebon et al. (2022). For example, practical advantages are related to the possibility of using the continuous presentation of several stimuli in a relatively short recording session. Importantly, it offers an objective definition of the targeted responses: based on the frequency of the stimulation, SS-EPs are expected at a specific narrow frequency band (Zhou et al., 2016). Previous studies on newborns and infants applied frequency-tagging paradigms with periodic visual stimulation to investigate face and object processing (e.g., de Heering and Rossion, 2015; Peykarjou et al., 2017; Buiatti et al., 2019), and with rhythmic speech sounds to investigate the tracking of transitional probabilities in the context of artificial grammar learning (Kabdebon et al., 2015; Choi et al., 2020; Fló et al., 2022).

Despite the growing interest in this approach, the synchronization of EEG oscillations with complex auditory exogenous rhythms are still relatively unexplored in infants. Using the same paradigm and methodology proposed by Nozaradan et al. (2011), a recent study has measured infants’ neural entrainment to simple rhythmic patterns (i.e., tone sequences), showing SS-EPs at a frequency corresponding to both beat and meter in infants as young as age 7 months (Cirelli et al., 2016). This study additionally provided preliminary evidence that such neural responses can be influenced by infant individual differences and their early musical experiences. To our knowledge, this approach including a frequency-tagging paradigm has never been applied to complex musical/speech stimuli in infancy.

**FIGURE 1:**
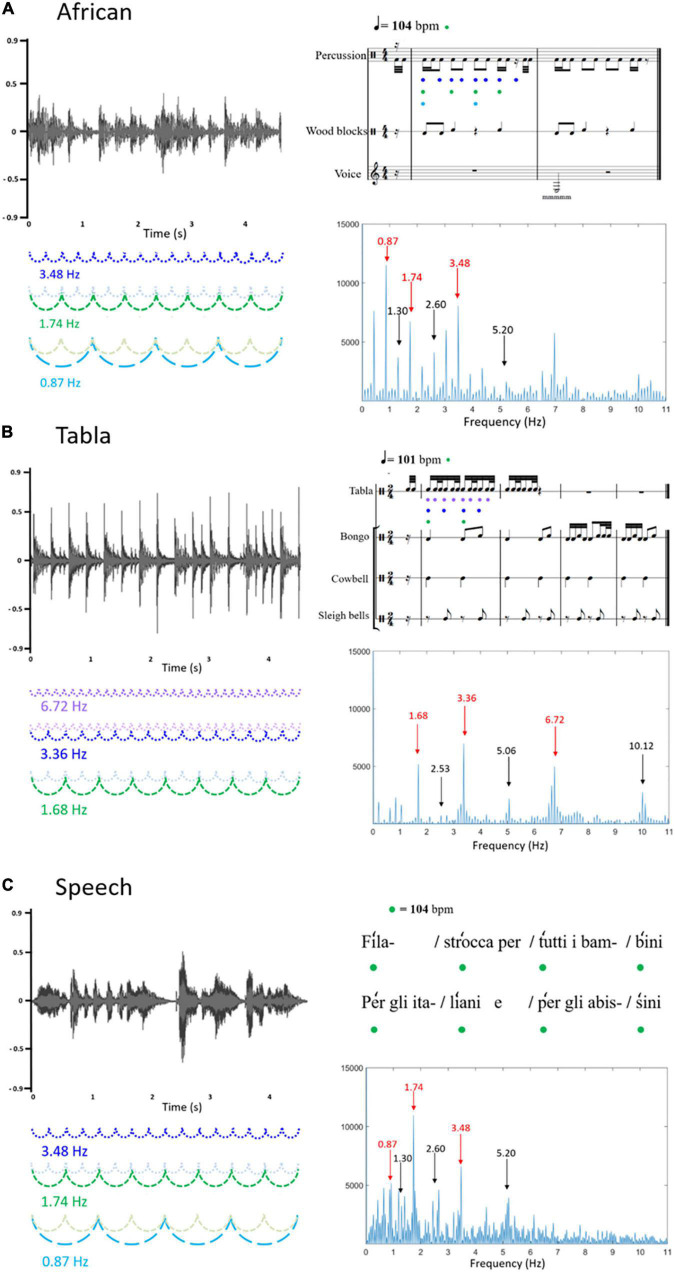
Graphical representation of the three sound stimuli: **(A)** African musical stimulus; **(B)** Tabla musical stimulus; **(C)** Speech stimulus. On the left, the three sound waveforms are reported (x-axis: time; y-axis: sound amplitude). On the right, the upper figures represent the rhythmic patterns, depicted in musical notation **(A,B)** or speech metric structure **(C)**; the bottom figures represent the frequency spectrums of the stimulus sound envelopes (*x*-axis: frequency; *y*-axis: magnitude). In the frequency spectrums, peaks at frequencies corresponding to the rhythm envelope are reported in red, whereas peaks at frequencies not corresponding to the rhythm envelope, considered as noise floor, are reported in black. Frequencies corresponding to the rhythm envelope are represented on the left, coupled with the sound waveforms, and on the upper right, coupled with musical notation or speech metric structure. In detail, it can be noted that for the African musical stimulus **(A)** frequency 3.48 Hz (represented in blue) corresponds to the fastest element detectable in the stimulus (matching with the quaver), frequency 1.74 Hz (represented in green) corresponds to the beat (104 bpm, matching with the crotchet), frequency 0.87 Hz (represented in light blue) corresponds to a chunk of notes (matching with the minim); for the Tabla musical stimulus **(B)** frequency 6.72 Hz (represented in lilac) corresponds to the fastest element detectable in the stimulus (matching with the semiquaver), frequency 3.36 Hz (represented in blue) corresponds to a chunk of notes (matching with the quaver), frequency 1.68 Hz (represented in green) corresponds to the beat (101 bpm, matching with the crotchet); the speech stimulus **(C)** was recorded to match with the African musical stimulus, with the metronome used to keep the beat set at 104 bpm (corresponding to frequency 1.74 Hz, represented in green).

In the present study, we investigated neural entrainment to complex music and speech stimuli in a sample of 8-month-old infants. Two different music rhythm patterns and a nursery rhyme with a regular and rhythmic pattern were selected based on their temporal envelope and their frequency spectrum of acoustic energy. Neural entrainment to the incoming rhythms was measured in the form of SS-EPs at frequencies corresponding to the rhythm envelope. By means of this methodology, the present study has a threefold aim. First and foremost, we aimed to investigate whether infants’ neural entrainment, already reported for simple rhythmic stimuli (Cirelli et al., 2016), is also present in response to much more complex stimuli. Second, since these stimuli were never used before, we piloted the experiment on adults: here we provide a developmental analysis to explore potential similarities and differences between infants’ and adults’ ability to entrain to the stimuli. Third, we analyzed the associations between infants’ measures of neural entrainment and the concurrent level of linguistic, cognitive and social-emotional development. This last aim rises from recent theoretical frameworks proposing entrainment of neural oscillations to external rhythmic stimuli as one of the mechanisms underlying speech and language (a)typical development (Ladányi et al., 2020; Fiveash et al., 2021). We expect to see in infants at least some sort of initial capability to entrain to the incoming complex auditory music and speech rhythms, even with some differences with respect to adults (i.e., more robust entrainment in adults than infants). In order to quantify the individual degree of entrainment, for each stimulus, we selected frequencies of interest based on the frequency spectrum of the stimulus sound envelope. As described in detail in Figure 1, the selected peaks corresponded to the fastest elements detectable in each stimulus and to more abstract chunks, corresponding to the beat and the meter. Finally, we expect that individual differences in the ability to entrain to these rhythmic features could be related to the overall infant developmental level, and specifically to language skills.

## Materials and methods

### Participants

Thirty-three families participated in this study. Infants were recruited at 6 months of age via local advertisements as part of a larger longitudinal study. The study was approved by the Medea Institute’s Scientific and Ethical Committees and all parents gave their written consent prior to testing. Infants were included if (1) both parents were Italian native speakers, (2) gestational age was ≥35 weeks and birth-weight was ≥2000 grams, (3) first-degree relatives had no certified diagnosis of intellectual deficiency or neurodevelopmental disorders. Data for this study were collected between 7 and 9 months of age. Since data from 8 children were rejected due to insufficient artifact-free trials in the EEG task, the final sample consisted of 25 infants (12 males; mean age = 8.00 months; in days, *M* = 240.16, SD = 19.13, min = 208, max = 279). Sample size was determined based on the previous SS-EP literature in similar populations (Kabdebon et al., 2015; Cirelli et al., 2016; Peykarjou et al., 2017; Choi et al., 2020; Fló et al., 2022). Information about parents’ and children’s daily exposure to music was collected, in order to better characterize the sample from this point of view. Mothers reported to spend on average 62.4 min per day (SD = 49.8) listening to music together with their child, while fathers reported to do it for 13.9 min on average per day (SD = 16.7). Moreover, 94.1% of the mothers and 62.5% of the fathers reported to be used to moving and “dancing” with their child following the rhythm of the music.

In addition to infants, a sample of 10 adults (8 females, mean age = 25.2 years; SD = 1.2; two left-handed) was recruited to provide a developmental comparison of neural entrainment between adults and infants.

### Clinical assessment

The Bayley Scales of Infant and Toddler Development – Third Edition (Bayley, 2006) were used as neuropsychological assessment. For the purpose of this study, the Cognitive, Language, and Social-emotional scales were administered. Considering the age range of our interest, the Cognitive scale mainly investigates sensory-motor development and the ability to explore and manipulate objects; the Language scale investigates receptive communication skills, such as the infants’ pre-verbal behavior and verbal comprehension, and expressive skills, such as pre-verbal communication (e.g., babbling and gestures) and early speech. The Social-emotional questionnaire is filled out by the caregiver and investigates the child’s interest and responsiveness toward people, objects and sounds and their communicative behavior. Scaled scores (*M* = 10; SD = 3) were calculated based on age.

### Electrophysiological recording

#### Stimuli

The stimuli consisted of three rhythmic patterns: two of them were instrumental music modulated at different rates and one was an Italian nursery rhyme with a regular and rhythmic pattern (mean length was 37.19 s, SD = 0.66). Figure 1 shows a graphical representation of the three different stimuli that were used.

The first music stimulus, henceforth called “African,” was created by extracting two meters of an African 4/4 rhythm (see Figure 1A), the average tempo was 104 beats per minute (bpm). Trials of approximately 37 s were created using Praat software package by repeating the extracted meters for eight times.

The second music stimulus, henceforth called “Tabla”, was created by extracting four meters of a Tabla composition (see Figure 1B), the rhythm was 2/4 and the average tempo was 101 bpm. The extracted segment was repeated 8 times in order to obtain the 37-s-long clip.

The “speech” stimulus was an eight-verse excerpt of an Italian nursery rhyme (“Girotondo di tutto il mondo,” Rodari, 1960). The rhyme was recorded by a native Italian female speaker; it was regularly spoken with exaggerated accents following a metronome set on 104 bpm in order to match the tempo of the first musical stimulus (African), see Figure 1C. The whole excerpt was repeated two times in order to obtain the 37-s-long clip.

Each 37-s-long trial was repeated 5 times in a pseudo-randomized order (no more than two trials of the same type appeared consecutively).

The temporal envelope of the three rhythm patterns was extracted using the Hilbert function implemented in MATLAB (Mathworks). A Fast Fourier Transform (FFT) was then applied in order to compute the frequency spectrum of acoustic energy. As represented in Figure 1, the frequency spectrum of acoustic energy varied across stimuli. For the African musical stimulus, frequencies of interest were: 3.48 Hz, corresponding to the fastest element detectable in the stimulus (matching with the quaver); 1.74 Hz, corresponding to the beat (104 bpm, matching with the crotchet); 0.87 Hz, corresponding to a chunk of notes (matching with the minim). For the Tabla musical stimulus, frequencies of interest were: 6.72 Hz, corresponding to the fastest element detectable in the stimulus (matching with the semiquaver); 3.36 Hz, corresponding to a chunk of notes (matching with the quaver); and 1.68 Hz, corresponds to the beat (101 bpm, matching with the crotchet). Finally, the speech stimulus was recorded with the beat set at 104 bpm, corresponding to frequency 1.74 Hz. Peaks at 0.87 Hz and 3.48 Hz were additionally selected as frequencies of interest to match this stimulus with the African musical stimulus.

#### Procedure

During EEG recording, infants were seated on their caregiver’s lap in a sound-attenuated and electrically shielded room and were entertained with silent toys. Both caregiver and the experimenter were given sound-isolating earphones which prevented them from hearing the auditory stimuli. In addition, the experimenter was instructed to make non-rhythmic movements while playing. The whole recording lasted approximately 10 min.

#### Data acquisition and analysis

##### Infants’ data

Electroencephalography (EEG) was recorded using a 60-channel HydroCel GSN net with an EGI recording system (Electric Geodesic, Inc.). Signals were recorded online with a sampling rate of 250 Hz, vertex as the online reference, and a 0.1–100 Hz online bandpass filter.

After recording, EEG data were exported to a MATLAB (Mathworks) compatible format and processed using EEGLAB (Delorme and Makeig, 2004) and the automated standardized pipeline MADE (Debnath et al., 2020). First, the 13 outermost channels were removed from analysis (Cantiani et al., 2016). Second, continuous EEG data were high-pass filtered at 0.5 Hz and low-pass filtered at 20 Hz. Third, bad channels were identified and removed using the EEGLAB plug-in FASTER (Nolan et al., 2010). As a fourth step, independent component analysis (ICA) was used to identify non-neural artifacts (e.g., ocular artifacts), and generic noise. As described in Debnath et al. (2020), the ICA procedure adopted in the MADE pipeline - including preparing a copy of the dataset by applying a 1 Hz high-pass filter, segmenting data into arbitrary 1-s epochs, removing noisy epochs, running ICA on the copied dataset and then copying ICA weights back to the original dataset - is an improved ICA decomposition that does not sacrifice low-frequency information or data that contain excessive noise. The Adjusted-ADJUST algorithm (Leach et al., 2020) was used to remove artifactual independent components (the average number of removed components was 5.16; SD = 3.13; range: 1–12). After ICA, data were epoched into 9-s segments starting from the onset of each stimulus, thus yielding 20 epochs for each stimulus. According to previous literature (Nozaradan et al., 2011; Cirelli et al., 2016), we removed the first second of each epoch to avoid the auditory evoked-potentials elicited by stimulus onset and because we expect entrainment to occur only after a few cycles of stimulation. Final epochs ranged then from + 1000 to + 9000 ms, with the baseline defined between 900 and 1000 ms. Residual artifacts were identified and treated as follows: epochs containing residual ocular artifacts (amplitude exceeding ±150 μV on frontal channels AF4, F2, Fz, Afz, F1, AF3) were removed, whereas all other non-ocular channels exceeding ±150 μV in a particular epoch were interpolated (within that particular epoch) using a spherical spline interpolation. Epochs with more than 10% of interpolated channels were rejected. Finally, data were re-referenced to the average of all the electrodes. Participants needed to have at least seven artifact-free epochs per stimulus to be included for analysis (excluded participants *N* = 8). The average number of artifact-free epochs per stimulus was: African (*M* = 15.56, SD = 2.68, range: 10–20), Tabla (*M* = 14.60, SD = 3.29, range: 9–20), Speech (*M* = 13.92, SD = 3.94, range: 7–20). A paired *t*-test revealed a significant difference in the number of artifact-free epochs for African vs. Speech [*t*(24) = 2.686, *p* = 0.013, FDR-adjusted *p* = 0.0390]. The average number of globally interpolated channels was 1.84 (SD = 0.89; range: 0–4).

Artifact-free trials were averaged and FFT was applied using Letswave6 (Mouraux and Iannetti, 2008). In order to remove the unrelated residual background noise, the magnitude of SS-EPs was calculated in relation to the amplitude of the frequency spectrum at surrounding bins. This was managed by extracting z-scores (i.e., the standard deviation relative to the distribution of the reference interval), considering neighboring bins from –0.15 to –0.09 Hz and +0.09 to +0.15 Hz around each frequency bin, corresponding to –5 to –3 and +3 to +5 bins around each frequency bin. Z-scores were applied instead of other baseline correction procedures (i.e., subtraction) because they are more easily interpretable (i.e., given the value it is possible to determine whether entrainment has occurred or not). As a further step, for each frequency peak of interest determined from the sound stimuli FFT (see section “Stimuli”), SS-EP magnitudes were extracted within a 0.2 Hz band centered on the frequency of interest (for example, for frequency 1.74, SS-EP magnitude was extracted considering the frequency range 1.64–1.84). In order to determine whether entrainment occurred or not at the specific frequencies of interest, we used two approaches. First, we interpreted z-scores as measures that include an estimate of the noise floor at the bins surrounding the frequency of interest (Peykarjou et al., 2017). Second, following Cirelli et al. (2016) we also calculated SS-EP magnitudes for frequencies that we did not expect to be relevant, based on the sound stimuli FFT. To select such frequencies for each sound stimulus, we computed the median between the first two frequency peaks of interest, and then computed the relative harmonics. Amplitudes at these peaks (three for each sound stimulus) are shown in Figure 1, and were considered as noise floor amplitudes. We believe that the results obtained applying this second method are even clearer and more interpretable than those obtained by the mere interpretation of z-scores, especially considering that the frequency stimulation with our stimuli cannot be as precise as the frequency stimulation with visual or less complex auditory stimuli.

SS-EP magnitudes computed for each channel were averaged in three clusters corresponding to left (channels AF3, F3, F5, FC5, F7, FT7), midline (channels F2, Fz, Afz, F1) and right (channels AF4, FT8, FC6, F8, F6, F4) frontal areas which were used in the analysis. Channels of interest were defined based on the previous ERP literature suggesting that frontal areas are involved in infants’ auditory processing (e.g., Choudhury and Benasich, 2011; Van Zuijen et al., 2012; Cantiani et al., 2016).

##### Adults’ data

The acquisition procedure was kept identical for adult participants, with the only difference concerning the use of 128-channel HydroCel GSN nets. Analytic procedures were also kept identical: the automated standardized pipeline MADE (Debnath et al., 2020) was used, since good performances of the included tools have been reported for adults as well (Leach et al., 2020). The only differences in the analytic procedures included: (1) no channels were removed from analysis *a priori*; (2) residual ocular artifacts were quantified as amplitude exceeding ±150 μV on frontal channels F10, AF8, AF4, FP2, FPZ, Afz, FP1, AF7.

The average number of adults’ artifact-free epochs per stimulus was: African (*M* = 18.10, SD = 3.31, range: 9–20), Tabla (*M* = 18.30, SD = 2.87, range: 11–20), Speech (M = 18.30, SD = 3.37, range: 9–20). No differences emerged between stimuli in the number of artifact-free epochs. The average number of globally interpolated channels was 7.10 (SD = 2.88; range: 1–10), whereas the average number of components removed following the ICA procedure was 31; SD = 7.75; range: 19–45.

Steady-state evoked potentials (SS-EP) magnitudes computed for each channel were averaged in three clusters corresponding to left (channels AF3, F3, F5, FC5, F7, FT7), midline (channels F2, Fz, Afz, F1) and right (channels AF4, FT8, FC6, F8, F6, F4) frontal areas which were used in the analysis.

#### Statistical analyses

Statistical analyses were run separately for infants’ and adults’ data. For each stimulus, we first ran a repeated measure ANOVA including noise (peaks of interests vs. noise floor), frequency (three levels for each stimulus), and laterality (left, midline, right) as within-subject factors. Greenhouse-Geisser-corrected p-values are reported when appropriate. Second, we ran paired-samples *t*-tests to compare SS-EP magnitudes at each of the frequencies contained in the sound stimulus and the most adjacent frequency not contained in the sound stimulus (noise floor): this follow-up analysis had the goal of identifying at which specific frequency entrainment occurred. Finally, for infants only, associations between SS-EP magnitudes at frequencies of interest significantly different from noise floor and concurrent measures of Cognitive, Language, and Socio-Emotional development were assessed using Pearson’s correlations. False discovery rate (FDR; Benjamini and Yekutieli, 2001) was applied to correct for multiple comparisons in the paired-samples *t*-tests (three comparisons for each stimulus) and Pearson’s correlations (twelve comparisons).

## Results

### Infants’ data

**FIGURE 2:**
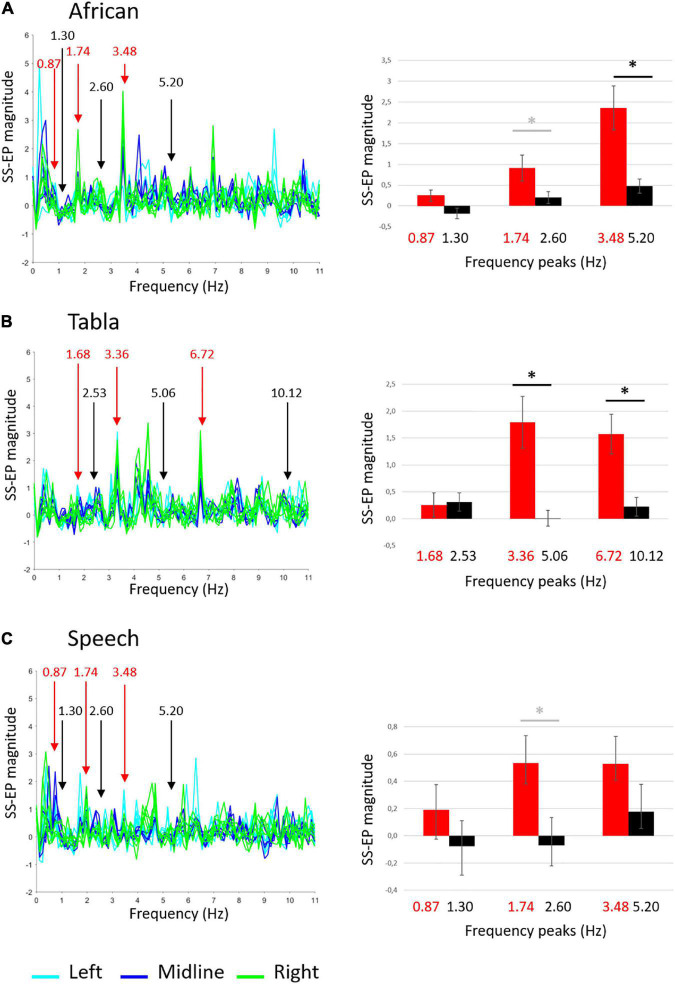
Group-level average SS-EPs for the three sound stimuli: **(A)** African musical stimulus; **(B)** Tabla musical stimulus; **(C)** Speech stimulus. On the left, butterfly plot of SS-EPs obtained applying the Signal-to-Noise correction (*x*-axis: frequency; *y*-axis: SS-EP magnitude). All EEG channels included in the clusters are plotted (Left: light blue lines, Midline: blue lines, Right: green lines). Peaks at frequencies corresponding to the rhythm envelope are highlighted in red, whereas peaks at frequencies not corresponding to the rhythm envelope, considered as noise floor, are highlighted in black. On the right, bar graphs (error bars indicate SEM) show the averaged SS-EP magnitude. Significant paired *t*-tests are indicated by black asterisks, whereas differences not surviving False discovery rate (FDR) correction are indicated by gray asterisks.

Group-level average SS-EPs for the three sound stimuli are shown in Figure 2. Descriptive statistics are reported in Table 1. SS-EP magnitudes significantly different from background noise are highlighted for both corrected (α < 0.005) and uncorrected (α < 0.05) thresholds.

**TABLE 1:** Descriptive statistics: Mean (Standard Deviation) on SS-EP magnitudes for frequencies of interest vs. noise floor separated for sound stimuli and cluster of channels.

	Peaks on the frequencies of interest				Noise floor amplitudes			
		Left	Midline	Right		Left	Midline	Right
	Hz	M (SD)	M (SD)	M (SD)	Hz	M (SD)	M (SD)	M (SD)
	0.87	0.47 (1.24)	0.27 (1.18)	–0.12 (0.56)	1.30	–0.24 (0.56)	–0.09 (1.48)	–0.21 (0.72)
African	1.74	0.90 (2.27)	0.76 (2.06)	1.07 (1.96)	2.60	0.24 (1.18)	–0.82 (0.81)	0.45 (1.38)
	3.48	**2.03** (2.25)	**1.96** (3.23)	3.07* (4.57)	5.20	0.44 (1.29)	0.26 (1.49)	0.73 (1.09)
	1.68	0.36 (1.69)	0.19 (1.68)	0.21 (0.91)	2.53	0.22 (1.08)	0.34 (1.46)	0.37 (1.12)
Tabla	3.36	**1.80** (2.81)	1.23 (2.02)	**2.34** (3.41)	5.06	0.15 (1.65)	–0.23 (0.87)	0.24 (0.85)
	6.72	**1.64** (2.36)	1.20 (2.25)	**1.80** (2.79)	10.12	0.17 (1.19)	0.35 (1.33)	0.14 (0.87)
	0.87	–0.18 (0.67)	0.58 (1.74)	0.17 (1.31)	1.30	–0.28 (0.47)	0.17 (1.85)	–0.11 (1.49)
Speech	1.74	0.89 (1.98)	0.44 (1.32)	0.26 (1.11)	2.60	0.20 (1.21)	–0.09 (1.18)	–0.31 (0.55)
	3.48	0.69 (2.38)	0.36 (1.02)	0.53 (1.00)	5.20	0.38 (0.85)	0.01 (1.28)	0.15 (0.85)

SS-EP magnitudes significantly different from background noise are highlighted for both corrected (α < 0.005, bold and *) and uncorrected (α < 0.05, bold only) thresholds.

The ANOVA carried out on the African musical stimulus revealed a main effect of noise, *F*(1,24) = 21.681, *p* < 0.001, *ŋ*^2^ = 0.475, a main effect of frequency, *F*(2,48) = 11.071, *p* < 0.001, *ŋ*^2^ = 0.316, and a significant interaction frequency × noise *F*(2,48) = 4.112, *p* = 0.035, *ŋ*^2^ = 0.146. Overall, as shown in Figure 2A, amplitudes relative to peaks of interest were higher than amplitudes relative to noise floor, and amplitudes were higher at higher frequencies than lower frequencies. Since we did not find any significant interactions including laterality, frequency, and noise, paired-samples *t*-tests were carried out to compare SS-EP magnitudes at each frequency of interest vs. noise floor averaging the three electrode clusters. Paired *t*-tests revealed that the difference between amplitudes in peaks of interests vs. noise floor was significant at the 3.48 Hz peak, *t*(24) = 3.610, *p* = 0.0030 (FDR-adjusted *p*-value), whereas the difference at the 1.74 Hz peak did not survive correction [*t*(24) = 2.148, *p* = 0.042, FDR-adjusted *p* = 0.0630]. These results suggest that infants reliably entrain to the fastest element detectable in the African stimulus (the quaver).

The ANOVA carried out the Tabla musical stimulus revealed a main effect of noise, *F*(1,24) = 17.533, *p* < 0.001, *ŋ*^2^ = 0.422, and a significant interaction of frequency × noise, *F*(2,48) = 6.240, *p* = 0.010, *ŋ*^2^ = 0.206. As shown in Figure 2B, paired *t*-tests averaging the three electrode clusters reveal that the difference between amplitudes in peaks of interests vs. noise floor was significant at the 3.36 Hz peak, *t*(24) = 3.356, *p* = 0.0039 (FDR-adjusted *p*-value) and at the 6.72 Hz peak, *t*(24) = 3.843, *p* = 0.0030 (FDR-adjusted p-value). These results suggest that infants reliably entrain to the fastest elements detectable in the tabla stimulus (the semiquaver and the quaver).

Finally, the ANOVA run on the Speech stimulus revealed only a main effect of noise *F*(1,24) = 6.478, *p* = 0.018, *ŋ*^2^ = 0.213 and a significant interaction frequency × laterality *F*(4,96) = 4.612, *p* = 0.006, *ŋ*^2^ = 0.139. Overall, as shown in Figure 2C, amplitudes relative to peaks of interest were higher than amplitude relative to noise floor. The interaction with laterality showed that at lower frequencies (e.g., 0.87 and 1.30 Hz) SS-EP magnitudes were higher at midline than at left, *t*(24) = -2.814, *p* = 0.010, whereas at higher frequency (e.g., 1.74 and 2.60 Hz) SS-EP magnitudes were higher at left than at right, *t*(24) = 2.414, *p* = 0.024. Since laterality affected SS-EP at frequencies of interest and at background noise similarly, paired *t*-tests were run averaging the three electrode clusters. They only reveal a difference between amplitudes in peaks of interest vs. noise floor at the 1.74 Hz peak, not surviving correction [*t*(24) = 2.342, *p* = 0.028, FDR-adjusted *p* = 0.0835]. These results suggest that infants show an overall effect of entrainment to the Speech stimulus, but they failed to reliably entrain to any specific frequencies.

### Adults’ data

Descriptive statistics for SS-EP magnitudes in all the three topographical clusters included in the analyses are reported in Table 2. SS-EP magnitudes significantly different from background noise are highlighted for both corrected (α < 0.005) and uncorrected (α < 0.05) thresholds.

**TABLE 2:** Descriptive statistics for the adults’ data: Mean (Standard Deviation) on SS-EP magnitudes for frequencies of interest vs. noise floor separated for sound stimuli and cluster of channels.

	Peaks on the frequencies of interest				Noise floor amplitudes			
		Left	Midline	Right		Left	Midline	Right
	Hz	M (SD)	M (SD)	M (SD)	Hz	M (SD)	M (SD)	M (SD)
	0.87	–0.37 (0.42)	-0.28 (0.58)	0.02 (1.09)	1.30	0.03 (0.73)	–0.33 (0.74)	–0.35 (0.40)
African	1.74	**2.23** (3.73)	2.88* (3.70)	2.78* (6.20)	2.60	-0.74 (0.67)	-0.40 (1.46)	-0.58 (0.29)
	3.48	0.42 (0.83)	**2.17** (3.83)	2.91* (5.33)	5.20	0.23 (0.99)	–0.04 (0.97)	0.28 (1.17)
	1.68	0.32 (1.24)	0.31 (0.94)	-0.11 (0.64)	2.53	0.35 (1.44)	0.73 (1.86)	–0.07 (1.16)
Tabla	3.36	1.36 (2.23)	3.90* (7.07)	2.92* (5.53)	5.06	**1.87** (2.21)	**1.69** (1.98)	0.59 (1.14)
	6.72	1.16 (2.04)	**2.43** (1.19)	2.80* (2.03)	10.12	–0.13 (0.99)	–0.42 (0.81)	–0.51 (0.71)
	0.87	0.43 (1.59)	–0.11 (0.74)	0.73 (2.22)	1.30	0.13 (0.79)	–0.08 (0.84)	0.07 (0.64)
Speech	1.74	0.97 (1.92)	**1.65** (3.17)	0.87 (1.73)	2.60	–0.07 (0.56)	-0.31 (0.69)	–0.14 (0.72)
	3.48	0.59 (1.70)	**2.37** (3.13)	**1.82** (1.75)	5.20	0.02 (0.77)	0.29 (0.91)	–0.22 (0.74)

SS-EP magnitudes significantly different from background noise are highlighted for both corrected (α < 0.005, bold and *) and uncorrected (α < 0.05, bold only) thresholds.

**FIGURE 3:**
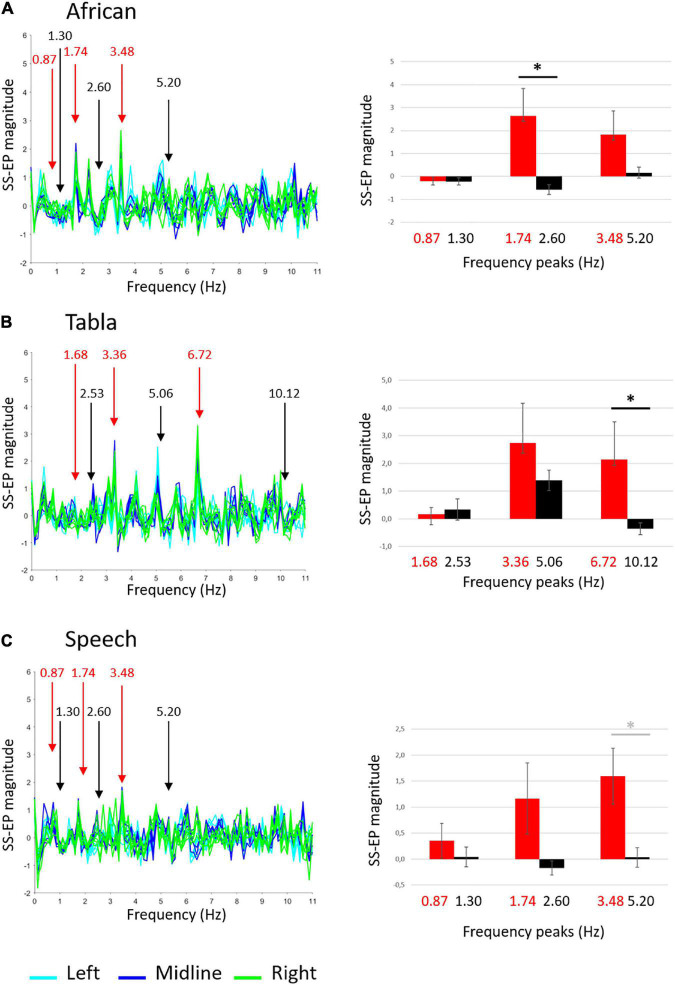
Adults’ group-level average SS-EPs for the three sound stimuli: **(A)** African musical stimulus; **(B)** Tabla musical stimulus; **(C)** Speech stimulus. On the left, butterfly plot of SS-EPs obtained applying the Signal-to-Noise correction (*x*-axis: frequency; *y*-axis: SS-EP magnitude). All EEG channels included in the clusters are plotted (Left: light blue lines, Midline: blue lines, Right: green lines). Peaks at frequencies corresponding to the rhythm envelope are highlighted in red, whereas peaks at frequencies not corresponding to the rhythm envelope, considered as noise floor, are highlighted in black. On the right, bar graphs (error bars indicate SEM) show the averaged SS-EP magnitude. Significant paired *t*-tests are indicated by black asterisks, whereas differences not surviving False discovery rate (FDR) correction are indicated by gray asterisks.

The ANOVA run on the African musical stimulus reveal a main effect of noise, *F*(1,9) = 7.419, *p* = 0.023, *ŋ*^2^ = 0.452, and a significant interaction frequency × noise *F*(2,18) = 4.252, p = 0.031, *ŋ*^2^ = 0.321. Overall, as shown in Figure 3A, amplitudes relative to peaks of interest were higher than amplitude relative to noise floor. Paired *t*-tests reveal that the difference between amplitudes in peaks of interests vs. noise floor was significant at the 1.74 Hz peak, *t*(9) = 3.101, *p* = 0.0381 (FDR adjusted p-value). These results suggest that adults reliably entrain to the beat of the African stimulus (the crotchet).

The ANOVA run on the Tabla musical stimulus reveal no significant main effects or interactions. As shown in Figure 3B, paired *t*-tests reveal a significant difference between amplitudes in peaks of interests vs. noise floor at the 6.72 Hz peak, *t*(9) = 4.950, *p* = 0.0024 (FDR adjusted p-value). These results suggest that adults reliably entrain to the fastest element detectable in the tabla stimulus (the semiquaver).

The ANOVA run on the Speech stimulus reveal a main effect of noise, *F*(1,9) = 7.491, *p* = 0.023, *ŋ*^2^ = 0.454. Overall, as shown in Figure 3C, amplitudes relative to peaks of interest were higher than amplitude relative to noise floor. Paired *t*-tests only reveal a difference between amplitudes in peaks of interests vs. noise floor at the 3.48 Hz peak, *t*(9) = 2.333, p = 0.045, not surviving FDR correction (FDR adjusted *p* = 0.1095). These results suggest that – similarly to infants – adults show an overall effect of entrainment to the Speech stimulus, but they failed to reliably entrain to any specific frequencies.

### Association with cognitive, language, and socio-emotional development

Associations between SS-EP magnitudes at frequencies of interest and concurrent measures of Cognitive, Language (combined measure including both expressive and receptive skills), and Socio-Emotional development were assessed using Pearson’s correlations. For each stimulus, we entered in the correlations SS-EP magnitudes at frequencies of interest that differ most from noise floor (i.e., 3.48 Hz for the African musical stimulus, 3.36 and 6.72 Hz for the Tabla musical stimulus, and 1.74 Hz for the Speech stimulus). Since basically no differences emerged concerning laterality, SS-EP magnitudes were averaged by topographical region.

**FIGURE 4:**
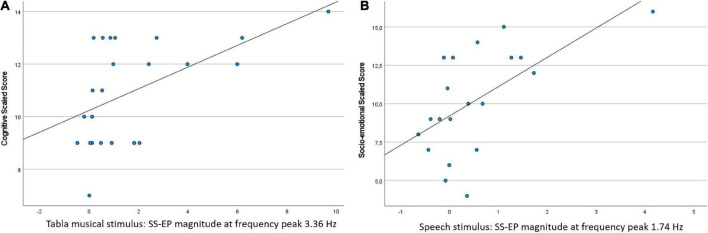
Scatterplots of the significant correlations between SS-EP magnitude in the neural entrainment task and concurrent standardized measures of development: **(A)** SS-EP magnitude in the Tabla musical stimulus (at frequency peak 3.36 Hz) is plotted against the cognitive scaled score; **(B)** SS-EP magnitude in the Speech stimulus (at frequency peak 1.74 Hz) is plotted against the socio-emotional scaled score.

Steady-state evoked potentials (SS-EP) magnitude for the Tabla musical stimulus at 3.36 Hz correlated with the Cognitive score, *r*(23) = 0.534, *p* = 0.0417 (FDR-adjusted p-value). Children with a greater SS-EP magnitude were characterized by higher cognitive scores. Additionally, SS-EP magnitude for the Speech stimulus at 1.74 Hz correlated with the Social-emotional score, *r*(20) = 0.604, *p* = 0.0417 (FDR-adjusted p-value). Children with a greater SS-EP magnitude were characterized by higher behaviors such as ease of calming, social responsiveness, and imitation play. Scatterplots are shown in Figure 4.

## Discussion

The main aim of this study was to investigate low-frequency neural entrainment in response to complex rhythmic stimuli in a sample of 8-month-old infants. Replicating the methodology used by previous studies with simple beat stimuli (Nozaradan et al., 2011, 2012; Cirelli et al., 2016), we successfully showed some degree of neural entrainment in young infants even for much more complex rhythmic stimuli. As already mentioned, one of the advantages of using SS-EP recording is that it offers an objective definition of what to expect (Kabdebon et al., 2022). Here, based on the frequency of the presented auditory stimuli, we expected SS-EPs at very specific narrow frequency bands. Interestingly, we have shown that for all the stimuli our sample of infants, as a group, presented overall higher SS-EP magnitude for frequencies of interest (selected based on the stimulus envelope) than for “noise floor” frequencies. These results are in line with recent works computing cortical/neural tracking to complex and naturalistic continuous stimuli, such as sung nursery rhymes or infant/adult directed speech (Kalashnikova et al., 2018; Attaheri et al., 2022a,b; Menn et al., 2022). These studies provided evidence for low frequency cortical tracking of the stimulus envelope, already at 4 months of age.

For the two music stimuli we found robust entrainment at specific frequencies, whereas for the speech stimulus we only found an overall effect against “noise floor.” It should be noted that in our study the music stimuli, although complex, were much more rhythmic than the speech stimulus. As described in section “Stimuli,” the nursery rhyme was pronounced following the beat of a metronome (hearable in the stimulus) with exaggerated accents. However, despite our efforts to make the speech excerpt as rhythmic as possible, the rhythmic entrainment to the physical properties of this kind of stimulus was not robust, probably because even young infants tend to process the speech stimuli differently from non-speech rhythmic stimuli. Similarly, studies on behavioral rhythmic sensorimotor synchronization show that infants aged between 6 and 16 months engage significantly more with rhythmic movements to music and non-speech rhythmically regular sounds than to speech (Zentner and Eerola, 2010). Overall, we found no hemispheric differences in the magnitude of SS-EPs, with the only exception being the speech stimulus, for which we found slightly more left-lateralized SS-EPs at higher frequencies. This left lateralization restricted to the processing of speech stimuli is not unexpected (Dehaene-Lambertz, 2017), and we interpret this finding as further evidence of different neural mechanisms underlying speech and non-speech processing. It should be noted that we retained a smaller number of artifact-free epochs for the Speech stimulus with respect to the African musical stimulus in infants. We cannot exclude that the smaller number of trials might explain the absence of a robust entrainment at specific frequencies for this stimulus. However, since we found the same results in adults, where no between-stimulus difference in the number of accepted trials emerged, we can rule out this possibility.

When comparing infants’ neural entrainment with that of a small sample of adults using the identical experimental stimuli and analytic methods, we found very similar results in terms of SS-EP magnitudes and frequencies to which neural entrainment occurred, in line with recent neural tracking evidence revealing overall “more developmental similarities than developmental differences” between infants and adults in the entrainment to sung nursery rhymes (Attaheri et al., 2022b). For the African musical stimulus, we found robust neural entrainment for both infants and adults, although at different frequencies: whereas infants entrained at 3.48 Hz, corresponding to the faster perceived elements at the rhythmic level (i.e., the quaver, as shown in Figure 1A), adults entrained at 1.74 Hz, corresponding to the beat (i.e., a less perceptual and more « abstract » feature of the stimulus). For the Tabla musical stimulus, we found robust neural entrainment for both infants and adults at the 6.72 Hz frequency, corresponding to the faster perceived elements at the rhythmic level (i.e., the semiquaver, as shown in Figure 1B). Interestingly, for this stimulus, we additionally found that children entrained at frequency 3.36 Hz, corresponding to the quaver. It should be noted that for this sound stimulus this more “abstract” level was also somehow detectable at the perceptual level (i.e., given by the combination of cowbell and sleigh bells). For the Speech stimulus, we found that adults, similarly to infants, did not show robust entrainment for any frequencies (nor for the perceptual beat of the metronome nor for more « abstract » beats). Overall, these results suggest that both adults and infants exhibit neural synchronization and tempo flexibility. This is partially contrasting with evidence reporting that, behaviorally, these skills do not emerge until the second year of age (Zentner and Eerola, 2010; Fujii et al., 2014; Yu and Myowa, 2021) and suggests that neural responses might be more sensitive than behavioral measures at a younger age.

Our last aim concerned the association between individual differences in infants’ measures of neural entrainment and the concurrent level of linguistic, cognitive, and social-emotional development. Contrary to our expectations, supported by theoretical frameworks proposing entrainment of neural oscillations to external rhythmic stimuli as one of the factors underlying speech and language development (Ladányi et al., 2020; Fiveash et al., 2021), we did not find any association with language skills. It should be noted, however, that here we only considered concurrent and very early language skills. Further studies should investigate such an association in a longitudinal perspective. Interestingly, associations were indeed found between neural entrainment to non-speech rhythmic stimulus – and more specifically between the ability to synchronize at the more « abstract » level (i.e., frequency 3.36 Hz) in the Tabla stimulus – and overall cognitive development. Additionally, we found that responses to nursery rhymes spoken by an adult female in infant-directed speech were associated to social-emotional development. Although not conclusive and in need of replication in larger samples, these correlations provide some preliminary evidence on the role of neural entrainment in development and support its functional interpretation.

Overall, the present study is a starting point for further investigation on the role of rhythm perception/synchronization in infancy (with more sensitive measures than behavioral ones) on later speech and language (a)typical development (Ladányi et al., 2020; Fiveash et al., 2021). In future research, the methodology proposed here could be coupled with novel analytic approaches including neural/cortical tracking performed in the time domain and thus might add convergent pieces of information. Both of these approaches seem appropriate to be used with infants at familial risk for Developmental Language Disorder and Dyslexia (e.g., Cantiani et al., 2016, 2019), since recent theories suggest that individual differences in this phenomenon could be one factor leading to atypical development trajectories of language acquisition found in these disorders (e.g., Goswami, 2011; Molinaro et al., 2016; Di Liberto et al., 2018). Furthermore, such measures seem appropriate for the investigation of the effect of early music/rhythmic training in infancy (e.g., Zhao and Kuhl, 2016; Dondena et al., 2021) in typical and atypical populations.

## Data availability statement

The raw data supporting the conclusions of this article will be made available by the authors, without undue reservation.

## Ethics statement

The studies involving human participants were reviewed and approved by Ethical Committee of the Scientific Institute IRCCS Eugenio Medea. Written informed consent to participate in this study was provided by the participants’ legal guardian/next of kin.

## Author contributions

CC, VR, MM, and CP designed the study. CC and CD run the experiment and collected the data. CC, CD, and CP analyzed the data. CC, VR, and CP interpreted the results. CC drafted the manuscript. All authors edited and revised the manuscript.
